# Comparison of Immersion and Portable Ultrasonic Housing to Quantify the Adhesive Bond Thickness and Sizing of Foreign Objects

**DOI:** 10.3390/ma17205111

**Published:** 2024-10-19

**Authors:** Nathaniel J. Blackman, Benjamin M. Blandford, David A. Jack

**Affiliations:** 1 Lonestar NDE, Waco, TX 76712, USA; nblackman@lsnde.com (N.J.B.); bblandford@lsnde.com (B.M.B.); 2 Department of Mechanical Engineering, Baylor University, Waco, TX 76798, USA

**Keywords:** non-destructive testing, ultrasound, immersion testing, portable inspection, bondline quantification, foreign object detection

## Abstract

High-performance materials, such as carbon fiber laminates, are costly to manufacture and are often used in demanding environments requiring the use of high-resolution non-destructive testing (NDT) methods to confirm the integrity of the parts. One NDT method that has shown promise for qualifying carbon fiber laminates is the use of immersion ultrasound with spherically focused probes. However, many parts may not be submersible in an immersion tank due to size or material constraints. These parts must be scanned with contact transducers with inferior resolutions or with expensive and messy systems such as bubblers. This research presents the use of a novel housing system that allows for the use of focused immersion transducers in an out-of-tank portable ultrasonic scanning application. This work presents a comparison between scans taken using a custom high-resolution immersion system and scans taken using the presented housing. There are a wide variety of potential inspection applications for this novel system, and the present work focused on two specific applications: the quantification of the spatially varying adhesive thickness in bonded carbon fiber laminates and the quantification of foreign object inclusions in carbon fiber laminates. The results presented show that scans using the portable housing are comparable in quality to scans performed using an immersion system. Specifically, both inspection approaches had an average error of 0.04 mm when quantifying the adhesive thickness of a bonded composite, and for the foreign object detection, the error in quantifying the dimensions of the embedded foreign object was 0.1 mm and 0.2 mm for the immersion system and the portable inspection system, respectively. The demonstration was performed in a laboratory setting, but a discussion is provided for the necessary improvements needed to extend the system for use in field applications.

## 1. Introduction

There is increasing pressure to achieve greater fuel efficiencies through the reduction of weight in both the automotive and aerospace industries [1]. The use of composite materials presents one solution. Carbon fiber composites, which have a high strength-to-weight ratio and anisotropic properties, are particularly desirable. Original Equipment Manufacturers (OEMs) such as Boeing in the aerospace industry and GMC in the automotive industry, along with many others, have opted to use carbon fiber composites prominently in designs, displacing heavier traditional materials.

Along with the adoption of carbon fiber composites comes the need for effective testing and qualification of parts. Traditional structural development testing is often destructive and, thus, quite costly. Non-destructive testing (NDT) methods are regularly used in manufacturing, where ultrasound is often selected due to its low cost and effectiveness, while in some applications, it can be made portable [2]. Ultrasound has been shown to be effective in composite investigations, such as characterizing the adhesive bond-line [3,4,5,6], delamination detection [7], and the identification of embedded foreign objects [8,9,10,11,12].

This presented work demonstrates the use of a novel housing system for immersion-style ultrasonic transducers. This housing allows for quality scans to be taken outside of an immersion tank and does not require the use of bubblers or water jets to immerse the probe in a water column. The demonstration is presented for two primary case studies: one for the quantification of spatial variations in the bondline thickness and the second of the quantification of the dimensionality of embedded foreign objects.

### 1.1. Spatially Varying Bondline Quantification for Use in Adhered Composites

It is often preferable to join composites with adhesives, bypassing fasteners and drilling within the composite to mitigate stress concentrations within the composite structure. Adhesive joints provide advantages in enhancing the load transfer between components and can create a seal between the joined components [13]. The final mechanical properties of the adhesive region are a function of both the quality and thickness of the bond [14,15,16,17,18]. Because of this dependence of the final part’s performance on the manufacturing process, NDT techniques are needed to quantify the quality and thickness of the adhesive region.

Previous researchers have used ultrasound to determine bond quality between metallic sheets [19], simultaneously measuring material properties (e.g., thickness, density, attenuation, and speed of sound) for a single material [20,21,22] and material thickness for multi-layered polymer components with homogenous layers [23,24]. However, there is little research on determining the adhesive thickness between two bonded composite laminates, such as the carbon fiber system studied in the present paper.

Poveromo and Earthman [5] explored the ability to create and analyze “kissing” bonds within adhesively bonded composite laminates. They used pulse echo ultrasound techniques to inspect the manufactured composites with a 5 MHz transducer and show the ability to detect the thickness of the adhesive layer. They studied both film and paste adhesive bonded samples and they identified the presence of a dis-bond for their samples with an adhesive thickness of 0.229 mm (0.009 in) for the film adhesive samples, where the nominal thickness of the adhesive film in the present study was 0.25 mm (0.010 in).

In a study by Stair et al. [25], the bondline between a carbon fiber laminate and an aluminum plate was investigated. They used a variety of different NDT ultrasonic techniques including contact, immersion, and phased array. Their ultrasound techniques were able to determine the presence of a dis-bond and focused on detectability requirements.

Current industry practices for obtaining adhesive thickness measurements require manufacturing precise calibration samples, ultrasonically scanning the samples, and storing the ultrasonic data from these known calibration samples. The stored data from the calibration are then used to confirm whether the manufactured components are within the acceptable tolerance. This is a time-consuming process and requires calibration samples for all material systems and permutations. Thus, there is a need for more robust NDT techniques capable of obtaining adhesive thickness measurements without calibration samples.

In the work by the current authors in [26], it was shown that ultrasound is capable of providing a spatially varying quantification of the adhesive layer thickness between two carbon fiber laminates when the speed of sound of the adhesive being used is known. The semi-automated technique was demonstrated on three uniform, consistent bond samples and two varying-thickness bond samples. The technique allows for quick and accurate thickness measurements with an absolute uncertainty of 0.05 mm (0.002 in) being achieved. The technique outlined in [26] was used in the current research for results from both an immersion system and the portable housing system.

### 1.2. Foreign Object Detection for Laminated Composties

Foreign objects find their way into composite laminates during the fabrication process [2]. Foreign objects are known to cause stress concentrations within composites, reducing the useful life of the component [27]. It is well documented that foreign objects and voids within a composite have a deleterious effect on the mechanical properties of the composite (see, e.g., [28,29,30]). In addition, foreign objects cause significant added expense to the use of carbon fiber laminates and are among the hardest defects to find as they are not a void or a high acoustic reflector but share some load transfer with the surrounding matrix and, thus, have a smaller acoustic echo to detect.

Previous researchers have demonstrated the capabilities of ultrasound to identify foreign objects in carbon fiber composites (see, e.g., [7,8,9,31,32]). Recent works published by Mohammadkhani et al. [10] and Ma et al. [11] presented more automated techniques for the detection of foreign objects based on the wavelet transform and signal correlation, respectively. Both works detected the depth of foreign objects quite accurately with errors of 2% and 1.8%, respectively. The algorithms differ in their ability to accurately characterize the size of foreign objects, where the algorithm of Mohammadkhani et al. [10] overpredicted the size of the Teflon object by 43.8% and the algorithm in Ma et al. [11] had errors in determining the edge-to-edge distance of the object, with the errors ranging from 0.475 mm to 0.175 mm.

In an earlier work by the present authors [12], a technique for measuring the diameter of circular foreign objects was demonstrated using the magnitude of the gradient. The technique was demonstrated on twelve carbon fiber samples with Teflon foreign objects of varying sizes placed at differing depths within the laminates. The average error in measuring the diameter of the foreign objects was 0.11 mm, while also demonstrating the ability to identify defects smaller than those in previously published research. This inspection technique and subsequent analysis are replicated in the current research and extended to compare the results of scans using the portable scanner with scans from a traditional immersion tank.

### 1.3. Technical Contributions of Present Work

The research presented in the current paper demonstrates the effectiveness of a novel field, portable ultrasonic housing system against that of a traditional c-scan-based immersion system ultrasonic inspection station. This portable housing allows for the scanning of parts from any alignment relative to the vertical while using high-resolution spherically focused immersion transducers that are typically limited to use in immersion scanning set-ups. The results presented are exclusively for the laboratory setting, but it is anticipated that future studies can expand the application space to more complex structures and industrially relevant geometries, a subject that is currently being pursued by the present authors. Scans taken using the housing are compared to those from an inspection system that fully submerges the part within water for both adhesively bonded composites and for composites with embedded foreign object debris. This is performed to demonstrate the resolution of the scans relative to that of cutting-edge immersion scan systems. The results presented indicate no difference in the adhesively bonded samples between the two systems but an error of 0.1 mm and 0.2 mm for the immersion system and the field portable ultrasonic inspection system, respectively.

There is room for improvement of the signal analysis aspects for both the bondline quantification and for the FOD detection, but this is outside of the scope of the present article. For example, the synchrosqueezed wavelet transform has been used successfully for the characterization of interlaminar damage [33] and could be readily extended to applications in thickness quantifications of the adhesive layer. Similarly, the use of wavelets for time-localized events [34] or the synchrosqueezed wavelet transform [35] can be used to identify the presence or absence of changes in the waveform at a prescribed time, which could be extended to identify the presence, or even the type, of FOD at various depths. The extension of the present work to further improve the signal analysis is left for a future study, and we keep the focus of the current paper on the comparison of the results between a laboratory immersion system and the portable ultrasonic inspection system.

## 2. Portable Ultrasonic Inspection System Housing

There are several commercially available options for using ultrasound outside of an immersion tank. Contact transducers suffer from limited spatial resolution and have a poor resolution at the front wall of the inspected component. Bubbler and water jet devices allow for the use of higher-precision immersion transducers. However, the flow of the water may alter or degrade the signal when scanning in a pulse-echo mode unless carefully controlled (see, e.g., [2]), and the area of inspection must be cleared of items that must stay dry. The current authors designed a portable housing system that addresses the issues of typical out-of-tank solutions and provides scans with a quality approaching that taken in an immersion tank.

**Figure 1:**
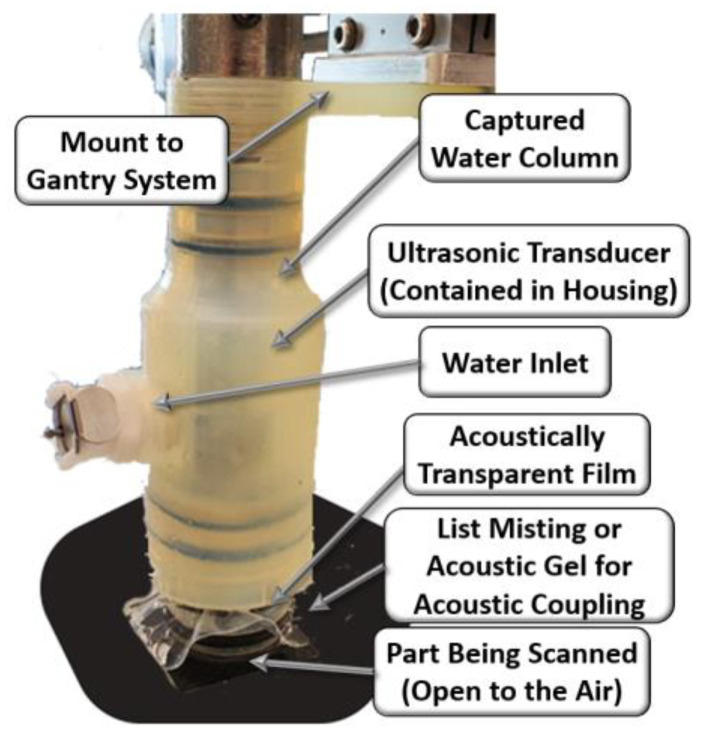
Image of the housing scanning a composite part.

A photograph of the housing system is shown in Figure 1. The housing has four primary components to its design. The first component is the search tube coupler, which holds the ultrasonic search tube and transducer in alignment with the housing. The coupler is threaded, which allows for the fine adjustment of the position of the transducer. This is a necessary component for inspecting and allows the operator to adjust the focal plane of the transducer prior to scanning. The second component is the sealed fluid chamber. By having a fully sealed chamber of water, this housing does not have the drawbacks that bubbler and squirter systems experience from moving water. The fluid within the chamber is easily filled using the inlet and then sealed during operation. The water has no significant movement and has no impact on the ultrasonic wave. In addition, by encasing the water, the relative orientation of the housing does not matter; thus, a scan could be performed upside down with no impact on the results. The third component of the device is the lens, an acoustically transparent film. The housing makes use of a thin elastomeric silicone as a lens material. The lens allows for the ultrasonic wave to pass through to the inspected part with minimal loss of energy or impact on the behavior of the waveform. The device also has a water inlet, which also serves as an air exhaust, thus preventing any captured air from negatively impacting the inspection. Notice the presence of a transducer within the housing. In the present study, results from a 10 MHz spherically focused transducer with a nominal focal length of 37.5 mm are shown; the results were found to be acceptable.

**Figure 2:**
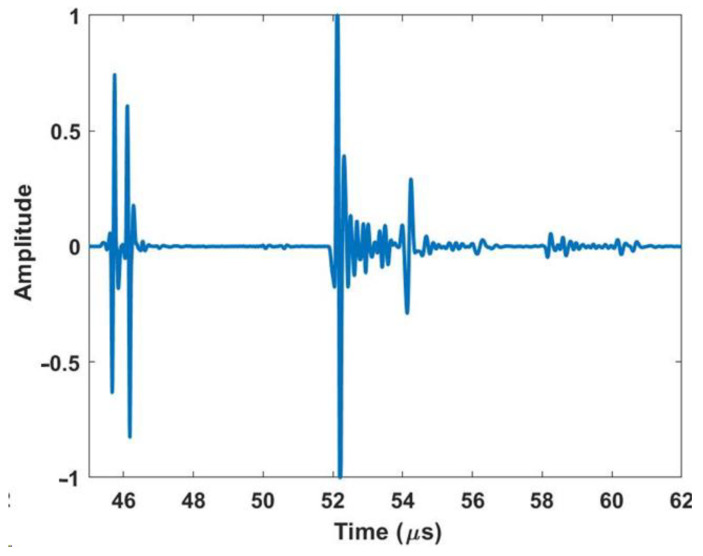
Representative a-scan of a composite part using the housing.

**Figure 3:**
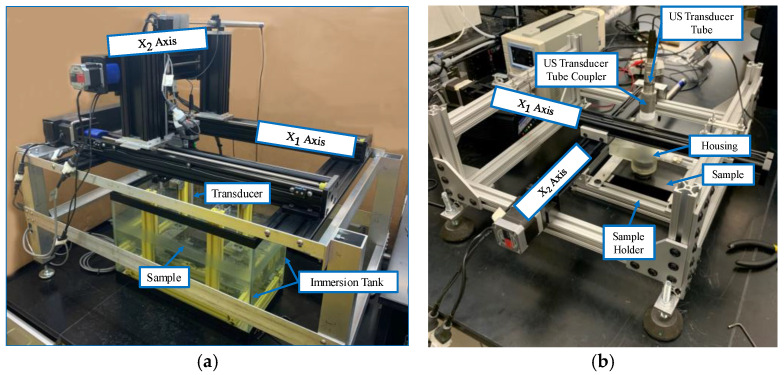
Ultrasonic inspection systems, (**a**) Representative immersion scanning system, and (**b**) Out of tank portable housing system.

Figure 2 shows a representative waveform captured using the ultrasonic housing showing the first reflection from the lens at approximately 46 µs, the waveform from the composite part occurs between 52 µs and 54 µs, and the first echo between the lens and the composite part occurs at approximately 58 µs. A thin layer of water from a mister or ultrasonic gel is needed between the lens and the part being scanned to complete the acoustic path. The last component of the system is the connection to a positioning system for scanning. In the current iteration shown of the portable housing in Figure 1, the housing is designed to interface with a 2-D rastering system made up of Velmex (Bloomfield, NY, USA) linear stages such as that shown in Figure 3a,b. The setup of the portable housing has some parallels to that of the immersion system, the most notable being that the transducer must be placed normal to the surface being inspected and then brought into focus. This is performed by leveling the part surface being scanned relative to the plane of scanning. In an immersion tank, this is often performed by subtle adjustments of the platen holding the part for each new part being inspected. Conversely, for the portable system, the focusing and alignment are performed for only the first part to be inspected by adjusting the feet and focal axis of the portable system and then locking it into place, using set screws in our case. There is no need to adjust the alignment when a new part is scanned or when a new region of a large part needs to be scanned. For example, we utilized the portable housing to inspect radomes and wing sections, neither of which can be scanned in an immersion tank. All that is required to setup the scan when moving from position to position is a light misting of water or acoustic gel on the surface of the part, a setup process that takes less than a minute and only requires a shop rag to clean the surface after inspection.

**Figure 4:**
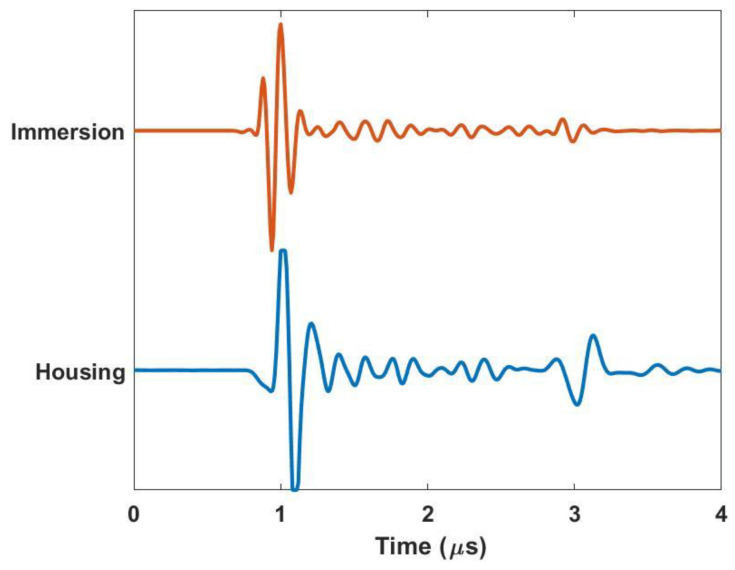
Comparison of a-scans for the immersion tank and housing inspection system.

Figure 4 demonstrates the similarity in fidelity between scans taken with the housing in a portable configuration and scans taken on a more traditional immersion system such as that shown in Figure 3a. The representative a-scans are of a simple composite laminate that is ~2 µs (~3.0 mm) thick and are performed using a spherically focused 10 MHz transducer with a nominal focal length of 37.5 mm. Notice that the time in the figure is shifted to be zero just before the front wall. Observe that the behaviors of the two waveforms are very similar. It is worth noting that for the present scan, a gain of 40 dB was used for the immersion tank, whereas a gain of 47 dB was used for the portable housing scan. This increased gain is due to the signal loss of the acoustic lens and the interfaces between the lens, water, gel, and part surface. There is a subtle shift in the nature of the initial wave at the front of the part at ~1 µs. This is expected as the acoustic wave takes a different path for the immersion system (transducer, water, part) relative to the portable housing (transducer, water, lens, gel/water, part). The behavior at the back wall of the sample is also of note, as the reflection occurring at approximately 3 µs is much greater relative to the front wall reflection for that of the scan taken with the housing. This is because the sample is being scanned in air as opposed to being submerged. This can be a potential benefit of the portable housing as compared to that of an immersion tank scan when the signal-to-noise ratio is a premium. The relative acoustic mismatch between air and the composite is much greater than between water and the composite.

## 3. Experimental Procedure

Carbon fiber composite laminates used in both studies were fabricated using a 3 K, 6 oz. plain-weave carbon fiber fabric from ACP Composites (Livermore, CA, USA). The INF 114 and INF 211 epoxy resin and hardener system from Proset (Bay City, MI, USA) was chosen for the fabrication of the composites. The samples were infused with the resin-hardener system using a Vacuum Assisted Resin Transfer Method (VARTM). In a companion study by one of the authors in his dissertation [26], similar studies were performed for fiberglass composite panels but without the detail presented in the current paper. Studies are ongoing by the present authors to investigate the practicality of the system’s usage for other carbon fiber systems, fiberglass systems, and also metallic systems, each of which will be presented in future works.

### 3.1. Adhesive Bondline Sample Preparation

**Figure 5:**
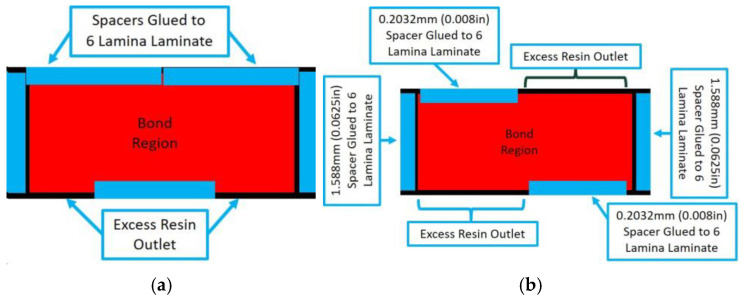
Bond configuration for (**a**) Uniform bonded adhesive spacer placement used for samples Bond 1, Bond 2, and Bond 3 and (**b**) Spacer placement used for Irregular Thickness Bond coupon.

Adhesive bonds were manufactured by adhering a 3 lamina laminate with a stacking sequence of [0/45/0] to a 6 lamina laminate with a stacking sequence of [0/45/0]_s_. The adhesive was a two-part epoxy purchased from Fibre Glast (Brookville, OH, USA). A total of 5 samples were fabricated, of which 3 samples had a uniform thickness and 2 samples had varying thickness. Curing was performed in two steps. The first step is the VARTM of the individual laminates, and the second step occurs after the adhesive is placed between two laminates. Spacers of three different sizes, 0.203 mm, 0.508 mm, and 1.59 mm, were used to create different thicknesses of the final fabricated coupons, namely, Bond 1, Bond 2, and Bond 3, respectively. The schematic showing the fabrication fixture for the uniform bonds is shown in Figure 5a with the spacer placement shown by the blue rectangles. Tooling was placed on both sides of the part and placed in a press during curing. A second type of sample, termed the Irregular Thickness Bond, was created using different placements of the spacers as shown in Figure 5b. Although the adhesive thickness is not truly random, the final surface geometry is a function of the holding pressure and is difficult to control precisely. To add to the irregularity of the adhesive geometry, clamps, rather than a uniformly distributed weight, were intentionally placed on the tooling surfaces. The key point is that consistency of the part is not relevant in the present context, and the variability is used to highlight the presented inspection method in characterizing spatial variations in the adhesive bondline.

### 3.2. Foreign Object Sample Preparation

This work made use of the same carbon fiber composite samples studied in [12]. Foreign objects were fabricated from thin sheets of Polytetrafluoroethylene (PTFE), measuring 0.05 mm in thickness. A Silhouette Cameo (Lindon, UT, USA) was used to cut three circles each of four different diameters from the PTFE sheet for a total of 12 circles. The designed diameters studied were 12.7 mm, 6.35 mm, 3.18 mm, and 1.59 mm. Prior to fabricating the carbon fiber samples, the foreign objects’ true size was determined via microscopy using a Keyence VR 3000 (Itasca, IL, USA), and these results are shown in Table 1. The accuracy of the presented results is greater than those reported in the table. The Keyence VR 3000 has a calibrated allowable error of 0.005 mm, and during calibrations before and after this present study, the error during calibration was undetectable, meaning it was less than 0.0005 mm. Based on an internal study of the roundness of the objects, we found that objects with diameters ranging from 2 to 10 mm had a roundness, defined as the ratio of 4π times the area divided by the perimeter, ranging from 0.985 to 0.995, where 1 would be perfectly circular. These twelve foreign objects were placed into twelve different carbon fiber composites during the layup process for a total of twelve individual coupons. Each foreign object was placed approximately in the center of a 50.8 mm × 50.8 mm sample prior to infusion with a 12 lamina layup of [0/30/60/0/45/0]_s_. Each sample was given a letter to designate the size of the foreign object and a number to designate the depth region of the foreign object. The letters A–D represent the four diameters of foreign objects, with A representing the largest, being 12.7 mm diameter objects, B the nominally 6 mm diameter objects, C the nominally 3 mm diameter objects, and D the nominally 1.5 mm diameter objects. The numbers correspond to the depth at which the objects were placed, where 1 indicates placement between the third and fourth plies, 2 indicates placement between the sixth and seventh plies, and 3 indicates placement between the ninth and tenth plies.

### 3.3. Ultrasonic Scanning Systems

Both the adhesively bonded laminates and laminates with foreign objects were scanned using the custom immersion system, depicted in Figure 5a, and again with the custom out-of-tank system built around the portable ultrasonic housing, depicted in Figure 5b. Both UT systems use Velmex translation stages for the translation of the ultrasonic transducer and can be interfaced with any portable ultrasound pulser/receiver. Both systems use a linear voltage displacement transducer for the $x_{2}$ axis, often termed the scan axis, and a linear encoder for the $x_{1}$ axis, often termed the index axis. All scans in this research were performed with a planar resolution of 0.2 mm between individual A-scans in both the $x_{1}$ and the $x_{2}$ directions. A 10 MHz spherically focused immersion transducer with a 38.1 mm nominal focal length was used for scanning the adhesively bonded samples with a pulse width of 50 ns and a peak voltage of 190 V. For the coupons with embedded foreign objects, a 7.5 MHz spherically focused immersion transducer with a 38.1 mm nominal focal length was used along with a pulse width of 60 ns and a peak voltage of 190 V. An Olympus Focus PX (Center Valley, PA, USA) system was used for both the driving of the transducer and the digitization of the resulting captured acoustic wave.

## 4. Results and Discussion

### 4.1. Adhesive Bondline Results

**Figure 6:**
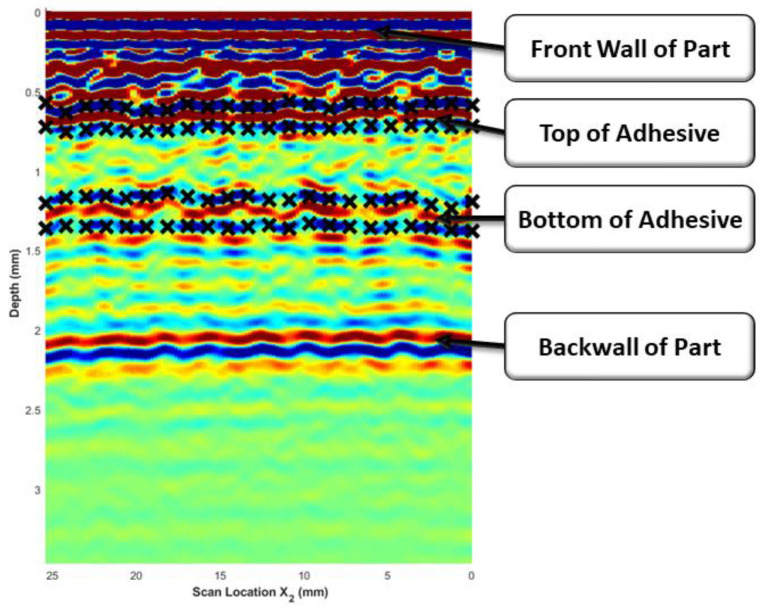
Representative B-Scan of an adhesively bonded laminate where the color corresponds to the normalized signal intensity with red being the highest intensity and blue being the lowest intensity. The black “×” symbols mark the manually bounded regions of the top and bottom of the adhesive region.

All scans of the adhesively bonded composites were analyzed using the methodology described by the current authors in a previous dissertation [26] to determine the adhesive thickness. The scope of the present study was not to develop a new algorithm for the waveform analysis but to utilize the methodology presented and detailed in [26] and compare the analyzed results between the immersion tank-collected results and the custom out-of-tank system results. The algorithm to determine the bondline thickness requires the user to manually select the reflections caused by the top and bottom of the bondline from a single b-scan. Figure 6 provides a representative example of the manually selected points for the top and bottom boundaries of the adhesive found at approximately 0.6 µs and 1.3 µs, respectively. A cubic spline fit was applied to bound the composite/adhesive interface, forming an initial estimate of the adhesive region as a function of $x_{2}$. This was then automated by tracking the peak along $x_{1}$, yielding a surface for both the top and the bottom of the adhesive. The automation method is fully discussed in [26].

**Figure 7:**
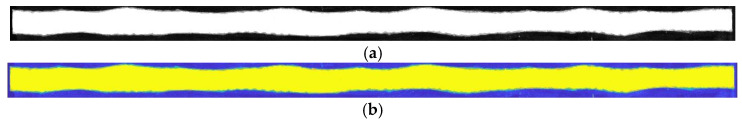
Stitched images of the 0.508 mm nominal bond along the $x_{1}=25.4$ mm cut line; (**a**) Image from the digital microscope (adhesive region is white) and (**b**) digitally processed image after filtering used for analysis (adhesive region is yellow).

**Figure 8:**
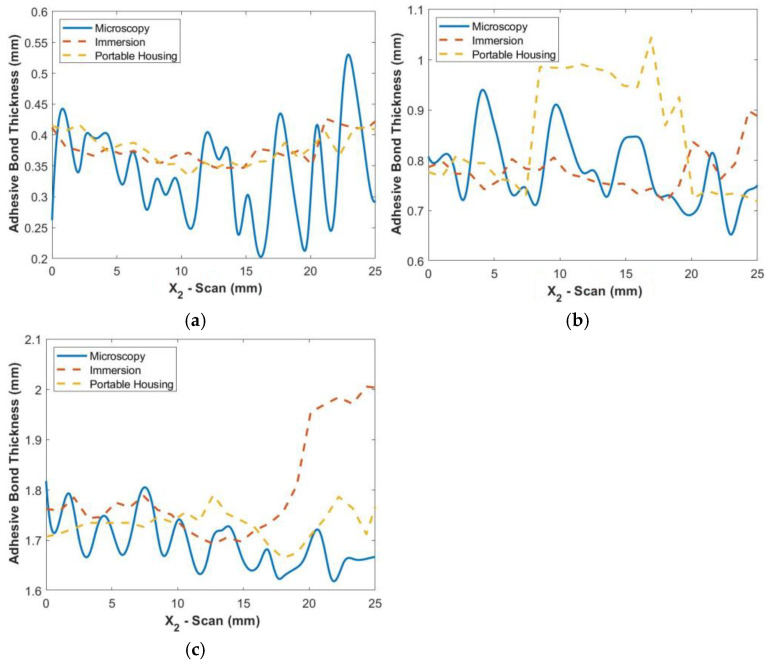
Comparison of microscopy thickness results with ultrasonic results from both immersion and portable scans along $x_{2}$ at the microscopy cut line $x_{1}=25.4$ mm for the uniform-thickness samples: (**a**) Bond 1, (**b**) Bond 2, and (**c**) Bond 3.

In order to verify the ultrasonic measurements, the adhesive samples were cut along the line $x_{1}=25.4$ mm after scanning and imaged using a Dino-Lite (Sanchung, Tai-Pei, Taiwan) digital microscope at a magnification of 100×. Images were taken using a polarizer to enhance the contrast between the adhesive region and the carbon fiber. At the magnification used, several images were required to capture the entire length of the cross-section, and the images were stitched together within Adobe Photoshop (San Jose, CA, USA). Figure 7a shows the stitched image of the bond from the digital microscope. Note that the polarization was adjusted to enhance the adhesive, in white, causing a high contrast relative to the region with carbon fiber tows, in black. This image was then smoothed in MATLB 2020a using a moving average filter, and then a spline fit of the surface was made as shown in Figure 7b. Pixels along the boundary were selected at the interface for the top and bottom of the adhesive bondline, and a cubic spline was fit to the points to provide a continuous function for later error quantification. Figure 8 shows the actual thickness along the cross-section for each of the three uniform bondline samples from microscopy imaging along with the thickness results from the immersion and portable scans. For both the immersion and portable scans, the identified thickness along $x_{1}=25.4$ mm follows the measured thickness from microscopy for the thinnest bondline sample, Bond 1. As can be noted in the figure, there is considerable oscillation in the adhesive thickness as observed in the microscope images, but this oscillation is significantly damped out from the ultrasonic data. This is expected as the ultrasonic signal is not of a single point but does carry with it a planar resolution similar to the length scale of 1/4th of a fabric tow; thus, the high oscillations observed in the microscope image would be damped out for the acoustic measurements. The feature to note is that the average amplitude is the effective adhesive thickness and is quantified in the present study along with the maximum point-wise difference. For example, in the Bond 1 sample, the peak difference between the ultrasonic inspection and the microscopic imaging results from the section sample is nominally 0.2 mm with an average difference less than 0.1 mm. In the case of the moderate thickness sample, Bond 2, the immersion system performed better, whereas the reverse was true for the thickest adhesive sample, Bond 3, and the portable housing system performed better. Regardless, the peak error for all three samples was 0.3 mm with an average error of less than 0.1 mm. Based on these results, there is a comparable accuracy for characterization between the portable housing and the immersion system. 

In terms of the present paper’s objective, to compare the results between the immersion system and the portable inspection system, the average difference, in terms of the absolute value, between the immersion and the portable system, was calculated. This was performed by taking pointwise the obtained thickness characterization of the adhesive from the immersion system $t_{imm}\left(x_{1},x_{2}\right)$ and the thickness characterization from of the adhesive from the portable housing system $t_{hou}\left(x_{1},x_{2}\right)$, and computing the average difference as

(1) $$\bar{d}=\frac{1}{x_{1,max}x_{2,max}}\int_{0}^{x_{2,max}}\int_{0}^{x_{1,max}}\left|t_{imm}\left(x_{1},x_{2}\right)-t_{hou}\left(x_{1},x_{2}\right)\right|dx_{1}dx_{2}$$

where the overbar on d represents the average difference between the two methods of characterization. Table 2 shows that there is a marginal difference between the two techniques between 0.02 mm and 0.06 mm. This value is noteworthy considering the expected wavelength of the acoustic wave within the sample. Using the classical definition that c=λf, where c is the speed of sound, approximately 3000 m/s for the laminates studied, and f is the frequency of the wave, nominally 10 MHz in the present study, a wavelength of nominally 0.3 mm is expected; thus, the difference between the two methods for the thickness quantification is less than the wavelength of the acoustic wave itself.

**Figure 9:**
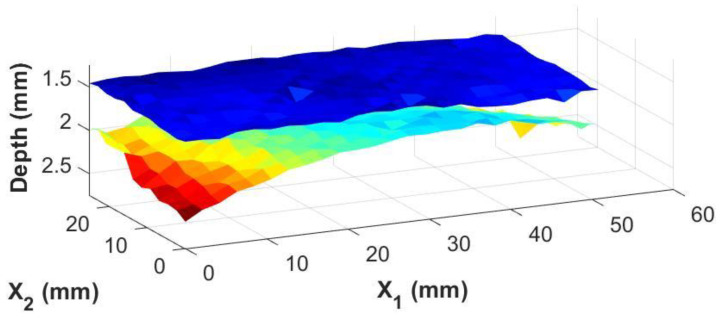
A 3-dimensional view of the top and bottom of the adhesive bondline from an immersion system where the color map represents the depth of the interface relative to the top surface of the coupon.

**Figure 10:**
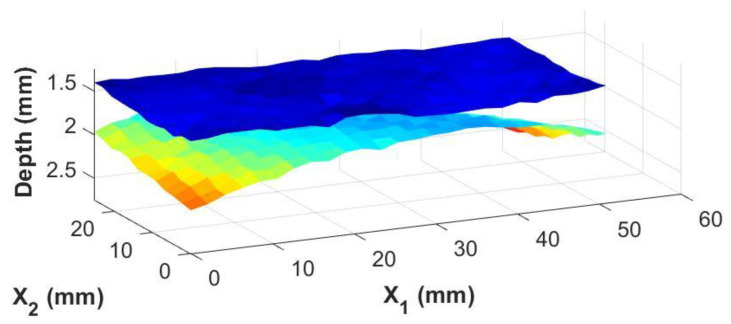
A 3-dimensional view of the top and bottom of the adhesive bondline from a portable housing system where the color map represents the depth of the interface relative to the top surface of the coupon.

The real advantage of the knowledge of the adhesive thickness at individual points is the ability to generate images of the adhesive/composite interfaces themselves. Using the irregularly shaped adhesive bond sample shown in Figure 5b, three-dimensional surface maps of the top and bottom interfaces between the bondline and the carbon fiber laminate skins were generated from the inspection date. These are shown in Figure 9 and Figure 10 for the results from the immersion system and the portable housing system, respectively. The figures show top and bottom interfaces, and the measurements between the two systems are very similar with subtle variations in the depth and contours of the interfaces similar to the magnitude of the error shown in Figure 8.

### 4.2. Foreign Object Debris Results

The analysis of the acoustic waveforms in the present study uses the technique previously developed and presented by the current authors in [12] to automatically extract the boundary perimeter of an embedded foreign object. The acoustic waveform intensity at each spatial location, defined as $F\left(x_{1,k},x_{2,l},t\right)$, is shifted in time as $\tilde{t}=t-t_{0}\left(x_{1},x_{2}\right)$, such that when $\tilde{t}=0$, the first front-wall echo occurs. After shifting, an averaged a-scan over the entire part is obtained at each depth into the part as

(2) $$F\left(\tilde{t}\right)=\frac{1}{x_{1,max}x_{2,max}}\int_{0}^{x_{2,max}}\int_{0}^{x_{1,max}}F\left(x_{1},x_{2},t\right)dx_{1}dx_{2}$$

where $x_{1,max}$ and $x_{2,max}$ are the length of the scan in the $x_{1}$ and $x_{2}$ directions, respectively. Using the average A-scan as a function of depth $F\left(\tilde{t}\right)$, the acoustic reflection corresponding to each of the n lamina can be identified. The times when these reflections occur are labeled $t_{n}^{*}$, and the time between peaks defined as $\Delta t_{n}^{*}=t_{n+1}^{*}-t_{n}^{*}$. For example, for a foreign object placed between the sixth and seventh lamina, such as in parts A2, B2, C2, and D2 from Table 1, the acoustic reflection from the sixth lamina will be chosen as the start of the gate, specifically $t_{6}^{*}$ in that case. Next, we use the maximum value of the signal within the gate, where the gate is defined as 75% of $\Delta t_{n}^{*}$, to avoid the reflection from the next lamina interface. This gated value, termed a C-scan, is defined as

(3) $$C\left(x_{1,k},x_{2,l}\right)=\underset{\tilde{t}\epsilon \left\{t_{n}^{*},t_{n}^{*}+0.75\times \Delta t_{n}^{*}\right\}}{\max}F\left(x_{1,k},x_{2,l},\tilde{t}\right)$$

**Figure 11:**
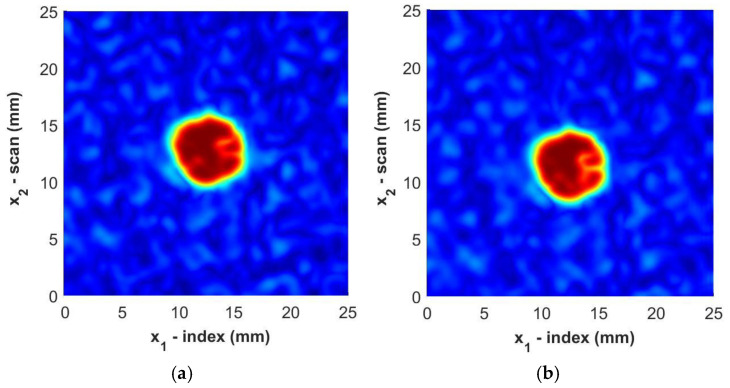
C-Scan images of Sample B3 from (**a**) immersion scan data and (**b**) portable housing scan data. Color represents the normalized intensity of the ultrasonic signal.

Next, a spatial gaussian filter is then applied to the data to smooth out boundaries as

(4) $$\tilde{C}\left(x_{1,k},x_{2,l}\right)=\frac{1}{2\pi \sigma_{x_{1}}\sigma_{x_{2}}}\int_{-\infty }^{\infty }\int_{-\infty }^{\infty }e^{\frac{{-\left({\tilde{x}}_{1}-x_{1,k}\right)}^{2}}{2\sigma_{x_{1}}^{2}\;}}e^{-\;\frac{{\left({\tilde{x}}_{2}-x_{2,l}\right)}^{2}}{2\sigma_{x_{2}}^{2}}}C({\tilde{x}}_{1},{\tilde{x}}_{2})\;d{\tilde{x}}_{1}d{\tilde{x}}_{2}$$

where $\sigma_{1}$ and $\sigma_{2}$ are the spread of the smoothing function and are selected to be nominally 0.3 mm in the present study. Next, a spatial interpolation is performed to densify the data set between the discrete points $\left(x_{1,k},x_{2,l}\right)$ to $\tilde{C}\left(x_{1,K},x_{2,L}\right)$, where the indices $\left(k,l\right)$ are mapped to $\left(K,L\right)$. In the present study, a five-fold densification was found to be adequate. An example of the total processed data is shown in Figure 11, with the immersion scan data in Figure 11a and the portable housing scan in Figure 11b. Notice that the weave can be seen in both figures, but it is not as striking as it would be without the filtering and, thus, the FOD contrast is highlighted with minimal irregularities from the reflections from the surrounding fabric weave. It is worth noting in the figure that the FOD identified is visually similar between the two inspections.

**Figure 12:**
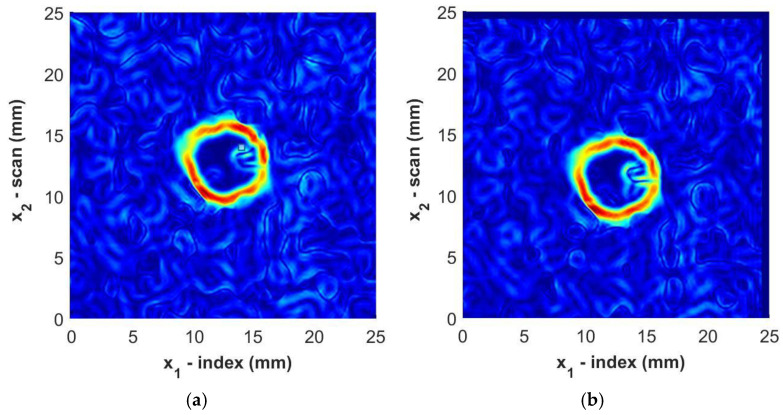
Magnitude of the gradient for Sample B3 from (**a**) immersion scan data and (**b**) portable housing scan data. Color represents the normalized intensity of the ultrasonic signal.

Next, the magnitude of the gradient of the C-scan $\tilde{C}\left(x_{1,K},x_{2,L}\right)$ is defined as

(5) $$\nabla \tilde{C}\left(x_{1,k},x_{2,l}\right)=\sqrt{{\left(\frac{\partial \tilde{C}\left(x_{1,k},x_{2,l}\right)}{\partial x_{1}}\right)}^{2}+{\left(\frac{\partial \tilde{C}\left(x_{1,k},x_{2,l}\right)}{\partial x_{2}}\right)}^{2}}$$

where the partial derivatives are evaluated using a simple central-difference method. The result for one sample, Sample B3, is shown in Figure 12 for the immersion tank and the portable housing inspection. Observe how the perimeters of the foreign objects are clearly visible in the figure regardless of the inspection method. The boundary of the foreign object is selected to be the local maxima of the gradient while tracing in a clockwise fashion about the centroid of the identified region of the foreign object.

The process outlined above is repeated for all twelve specimens listed in Table 1, and the results for the obtained area are provided in Table 3. In the table, the area from the microscopy results of the FOD prior to fabrication are shown along with the area obtained using the ultrasound inspection $A_{UT}$ from both the immersion testing as well as from the portable housing system. In the table, the system yielding the best accuracy as compared to the true area is highlighted in bold font. Notice that that 7 of the 12 results favor the immersion system whereas 5 of the results favor the portable system.

The area obtained from the analysis of the ultrasound inspection, $A_{UT}$, is converted to a length measurement through the effective diameter as

(6) $$\hat{d}=\sqrt{4A_{UT}/\pi }$$

The effective diameter allows one to state the resolution of the measurement using a base unit of length. The results for the diameter measurements from the various inspection methods are shown in Table 4. The results in Table 3 from the area from the two ultrasound systems are presented to the second digit after the decimal place. In an internal study, we found this value to be repeatable to within 0.05 mm^2^ when reanalyzing the data sets, which were correlated with a range of variability in the diameter at the third significant digit after the decimal place for the results presented in Table 4. As can be seen, the typical error is quite small and in all cases less than 0.75 mm with an average error of 0.11 mm for the immersion system and 0.21 mm for the portable system. Although the immersion system yields a higher accuracy, the portable system has an error close to that of the step size of the inspection itself.

## 5. Conclusions

A portable housing inspection system using an immersion quality ultrasonic transducer is presented. The purpose of this study was to provide a comparison of the ability to characterize internal features from the presented portable housing system and a standard immersion system. This study investigated two different types of internal features. The first is a through-thickness feature, specifically the adhesive bondline thickness between two carbon fiber composite structures. The second feature investigated is planar orthogonal to the incident inspection probe, specifically a manufacturing induced flaw of an embedded foreign object. The former demonstration quantifies the through-thickness accuracy, which in future studies could be extended to include features such as delaminations, planar voids, or impact damage depth. The latter study demonstrates the planar accuracy, which could be extended in future studies to features such as planar porosity, delamination area quantification, impact damage area, or dis-bonding. The results are compared between the two methods, and the results show that the use of the portable housing has little impact on the quality of the scan data. For example, the adhesive thickness measurements had an absolute average area across the scan region of 0.04 mm for all samples tested, regardless of the inspection system. For the inspections of the samples with embedded foreign objects, the average error was 0.21 mm from the portable inspection system and 0.1 mm from the immersion system. Both numbers are quite close to the step size of the inspection itself of 0.1 mm, but more importantly, are an order of magnitude lower than current industry systems that identify feature sizes to within 2 mm or as low as 1 mm. The novelty of this work is that the housing enabling out-of-tank inspections has little impact on the quality of the ultrasonic scan with respect to spatial measurements but allows for the freedom to perform scans on objects without the need for submerging the part in water. Specifically, the portable housing presented is capable of scanning parts that are not able to be submerged at any orientation relative to the surface. Unlike existing solutions, the housing does not require a water jet to provide coupling and provides the ability to obtain high-resolution scan data that cannot be provided by contact transducers. This device could enable high-quality ultrasonic measurements in both manufacturing and in-service environments providing greater detail for both technicians and engineers to assess the quality, potential performance, and safe use of components.

The present inspection system, shown in Figure 3b, is designed for use in the laboratory to allow for a comparison between the immersion scan inspection results from the system shown in Figure 3a and the results of the out-of-tank inspection system in the same laboratory environment. To extend the system for use in the field, demonstrations of the accuracy of the system over the range of temperatures and humidity conditions would be required for a prolonged conditioning of both the sample and the system to confirm operability within harsh environments. A challenge will be in forming the baseline of truth as immersion systems cannot operate in the harsh conditions required of field portable systems. As the current system utilizes water, the temperature bounds must be kept within the liquid regime without a change to a new couplant. Similarly, consideration must be given to the safe operability of the transducer itself as the electronics are susceptible to damage at elevated temperatures. The present authors are developing a field portable system as part of our ongoing studies, and our biggest challenge appears to be the gantry system’s ability to operate in harsh environments over prolonged periods. This is good as it is a problem that can be solved by a system redesign, whereas if the problems were the housing or acoustic membrane, further research would be required for deployment. The housing has been modified in related studies by the authors to interface with other scanning structures such as a robotic manipulator allowing for the scanning of more complex geometries. The authors look forward to future works presenting their observations on the conversion of the laboratory scale system to a field, portable system.

## 6. Patents

The following patents are correlated to the presented work.

System and Method for Real-Time Visualization of Defects in a Material—Visualization and GUI. D.A. Jack, B. Blandford, and N. Blackman. Issued April 2023, Patent Number 11,619,611.

System and Method for Real-Time Visualization of Defects in a Material—Bondline Thickness. D.A. Jack, B. Blandford, and N. Blackman. Issued June 2023, Patent Number 11,686,707.

System and Method for Real-Time Visualization of Defects in a Material—Foreign Object Detection. D.A. Jack, B. Blandford and N. Blackman. Baylor University. Issued August 2023, Patent Number 11,726,065.

System and Method for Portable Ultrasonic Testing. D.A. Jack, B. Blandford, N. Blackman, K. Garrett, and D. Smith. Issued January 2024, Patent Number 11,860,131.

## Figures and Tables

**Table 1:** Microscopy results for PTFE foreign objects.

Foreign Object Label	Diameter (mm)	Area (mm^2^)
A1	12.67	126.08
A2	12.55	123.70
A3	12.55	123.70
B1	6.08	29.03
B2	6.18	30.00
B3	6.10	29.22
C1	3.10	7.55
C2	3.13	7.69
C3	3.09	7.50
D1	1.52	1.81
D2	1.61	2.04
D3	1.52	1.81

**Table 2:** Comparison of the average difference between the immersion and portable housing system for adhesive thickness characterization.

Sample	Bond 1	Bond 2	Bond 3	Irregular Bond
d (mm)	0.02	0.04	0.03	0.06

**Table 3:** Comparison of equivalent diameters for all foreign objects between microscopy and ultrasonic scans from the immersion tank and portable housing. The more accurate measurement is in bold font.

Foreign Object	Microscopy Area (mm^2^)	Immersion Area (mm^2^)	Housing Area (mm^2^)
A1	126.08	**132.51**	141.44
A2	123.70	127.75	**123.49**
A3	123.70	**124.73**	126.74
B1	29.03	**30.81**	33.29
B2	30.00	**30.49**	32.31
B3	29.22	28.43	**28.70**
C1	7.55	**8.41**	9.85
C2	7.69	**7.78**	9.60
C3	7.50	7.53	**7.48**
D1	1.81	**1.82**	1.76
D2	2.04	1.87	**2.03**
D3	1.81	1.48	**1.80**

**Table 4:** Comparison of effective diameter $\hat{d}$ measurements for foreign objects between microscopy, immersion tank scans, and portable housing scans. The more accurate measurement is in bold font.

Foreign Object	Microscopy $\hat{d}$ (mm)	Immersion $\hat{d}$ (mm)	Error Immersion (mm)	Housing $\hat{d}$ (mm)	Error Portable (mm)
A1	12.67	**12.99**	**0.32**	13.42	0.75
A2	12.55	12.75	0.20	**12.54**	**0.01**
A3	12.55	**12.60**	**0.05**	12.70	0.15
B1	6.08	**6.26**	**0.18**	6.51	0.43
B2	6.18	**6.23**	**0.05**	6.41	0.23
B3	6.10	6.02	0.08	**6.05**	**0.05**
C1	3.10	**3.27**	**0.17**	3.54	0.44
C2	3.13	**3.15**	**0.02**	3.50	0.37
C3	3.09	3.10	0.01	**3.09**	**0.00**
D1	1.52	**1.52**	**0.00**	1.50	0.02
D2	1.61	1.54	0.07	**1.61**	**0.00**
D3	1.52	1.37	0.15	**1.51**	**0.01**

## Data Availability

The data presented in this study are available on request from the corresponding author.
